# Molecule–Environment Embedding with Quantum Monte Carlo: Electrons Interacting with Drude Oscillators

**DOI:** 10.1021/acs.jctc.5c00108

**Published:** 2025-04-30

**Authors:** Matej Ditte, Matteo Barborini, Alexandre Tkatchenko

**Affiliations:** † Department of Physics and Materials Science, University of Luxembourg, L-1511 Luxembourg City, Luxembourg; ‡ HPC Platform, University of Luxembourg, L-4364 Esch-sur-Alzette, Luxembourg

## Abstract

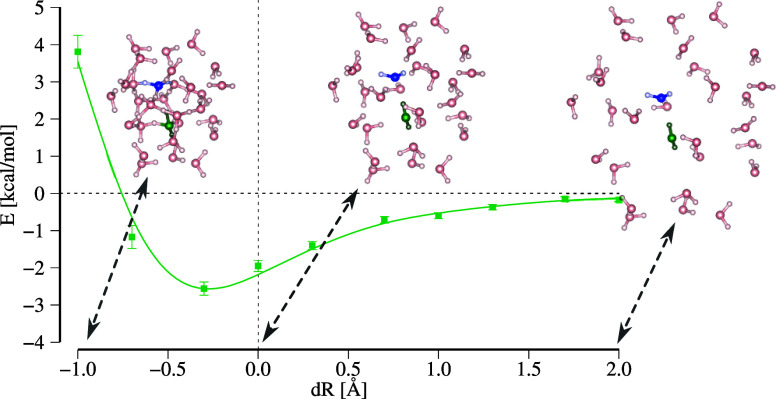

We present a comprehensive investigation of the El-QDO embedding method [*Phys. Rev. Lett.***131**, 228001 (2023)], where molecular systems described through the electronic Hamiltonian are immersed in a bath of charged quantum harmonic oscillators, i.e., quantum Drude oscillators (QDOs). In the El-QDO model, the entire system of electrons and drudons—the quantum particles in the QDOs—is modeled through a single Hamiltonian which is solved through quantum Monte Carlo (QMC) methods. We first describe the details of the El-QDO Hamiltonian, of the proposed El-QDO ansatz, and of the QMC algorithms implemented to integrate both electronic and drudonic degrees of freedom. Then we analyze short-range regularization functions for the interacting potential between electrons and QDOs in order to accurately treat equilibrium and repulsive regions, resolving the overpolarization error that occurs between the electronic system and the environment. After benchmarking various regularization (damping) functions on the cases of argon and water dimers, the El-QDO method is applied to study the solvation energies of the benzene and water dimers, verifying the accuracy of the El-QDO approach compared to accurate fully electronic ab initio calculations. Furthermore, through the comparison of the El-QDO interaction energies with the components of Symmetry-Adapted Perturbation Theory calculations, we illustrate the El-QDO’s explicit many-body treatment of electrostatic, polarization, and dispersion interactions between the electronic subsystem and the environment.

## 1 Introduction

The modeling of many important chemical and physical phenomena requires the accurate description of a manifold of energetic, spatial, and temporal scales, that typically cover several orders of magnitude.^1−3^ To efficiently treat these different scales, the last decades have seen the development of several hybrid computational embedding techniques,^1,3^ that can simultaneously tackle partitioned subsystems with varying levels of computational accuracy, paving the way to a feasible, yet accurate representation of important phenomena in molecules and materials.

A first group of embedding methods is characterized by a combination of quantum mechanics and molecular mechanics (QM/MM) methods, developed by the Nobel Prize winners, Karplus, Levitt, and Warshel among others.^4^ These methods split the chemical or physical systems into an environment described via classical force fields (FF) and a target subsystem described at the quantum level, usually through density functional theory (DFT).^1,3^ QM/MM methods have been successfully applied to treat a wide variety of phenomena including functional materials,^5,6^ catalysis,^7−10^ biochemical systems,^11−16^ enzymes,^17^ proteins,^18^ DNA^19,20^ and also Raman spectroscopy.^21,22^

A second group is composed of explicit QM/QM methods, where both fragments are treated quantum mechanically, using different levels of accuracy and thus different computational costs. Here, we can include both Green’s function methods for studying spectroscopic and thermal quantities like dynamical mean-field theory^23^ or self-energy embedding theory^24^ and the wave function/density-based methods for studying ground state properties like density matrix embedding theory^25^ or DFT embedding.^26−28^ Also, the QM/QM methods have been applied to a wide range of different systems and phenomena, including periodic graphene,^29^ catalysis,^30−35^ spectroscopy,^36−39^ defects,^40^ surfaces,^41,42^ strongly correlated states in materials,^43^ perovskites,^44,45^ complex oxides,^46^ nickelates^47^ and a variety of other real materials.^48^ Furthermore, embedding models for solvation include the Polarized Continuum Model (PCM),^49^ the Surface Generalized Born model (SGB),^50,51^ and the Conductor-like Screening Model (COSMO),^52^ and the Reference Interaction Site Model (RISM)^53,54^ together with many other specific polarizable force-fields.^55,56^

In this work, we present further improvement of a recently introduced quantum embedding approach,^57^ in which a molecule described through the electronic Hamiltonian is embedded into Coulomb-coupled charged quantum harmonic oscillators, i.e., quantum Drude oscillators (QDOs).^58−60^ The QDOs are used to describe the long-range response properties of the real atomic environment, with significantly reduced degrees of freedom when compared to the fully electronic description.^2,60−63^ The QDOs are parametrized to reproduce the leading order polarization and dispersion coefficients of real atoms/molecules exactly while approximating the higher-order coefficients (e.g., noble gas atoms or water molecule).^60,61,64−66^

Coulomb-interacting QDOs,^57,59,61,64,67,68^ as a coarse-grained model of atomic systems, have already been used within the framework of diffusion Monte Carlo (DMC) and path integral Monte Carlo (PIMC) to study the dispersion interactions in noble gas dimers,^60^ solid^64^ and fluid xenon.^61^ Another successful application is a QDO-based model of the water molecule,^69,70^ which was also applied to study liquid and solid water dynamics.^67,71^ A different framework in which QDOs have been exploited is that of the full configuration interaction (FCI) method, in which the oscillators are expanded in a basis set of Gaussian functions, and applied to prototype systems for dispersion interactions.^68^ Furthermore, the QDO model has been used to construct universal pairwise interatomic van der Waals potentials^72^ or to study dipole-bound anions via QDOs interacting with a single electron using perturbation theory.^73,74^ The QDO model is also employed in the many-body dispersion (MBD) method,^63,75,76^ in which the Coulomb interactions between oscillators are approximated by dipole interactions. This approximation leads to a quadratic Hamiltonian, which can be diagonalized and yields interaction energies that can be used as a many-body dispersion method within DFT.^2,77^

In the El-QDO embedding method^57^ the electronic subsystem and the QDOs’ environment, together with point charges, when necessary to represent electrostatic interactions from the environment,^60^ are described through a single comprehensive Hamiltonian. This approach treats the degrees of freedom of the electrons and drudons, thus explicitly including the many-body correlation effects between the electronic subsystem and the environment. The electronic and drudonic degrees of freedom are integrated using a collective variational ansatz within the framework of quantum Monte Carlo (QMC) methods.^78−80^ This new joint framework has also the advantage of overcoming difficulties and limitations in the construction of embedding methods between QMC and polarizable FFs^11^ or between QMC and other first-princples approaches, such as DFT.^81^

In our initial development of the El-QDO method,^57^ the effects of the solvent on the binding energies of the benzene dimer and on the singlet–triplet gap of ortho-benzyne, were studied by immersing these subsystems in an expanded cage of water molecules. These first results demonstrated how the El-QDO binding energies were compatible with explicit electronic calculations, improving with respect to the predictions from the classical embedding (El-FF with Lennard-Jones (LJ) potentials), and thus accurately capturing electrostatic, polarization, and dispersion interactions between the two quantum subsystems and the environment. The reason behind this accuracy was ascribed to the quantum nature of the QDOs interacting with the electronic system, which are able to include explicit instantaneous response of the environment and electronic subsystem through mutual correlations. The choice of working only with expanded water cages was also motivated by the need to avoid overpolarization effects that appear when the QDO environment and the electronic molecular subsystems are close to each other, as also happens in other QM/MM methods.^82^ At equilibrium and shorter distances between QDOs and electrons, Coulomb interactions induce nonphysical charge drifts that introduce divergences in the interaction energies.

In this work, apart from presenting the details of the El-QDO Hamiltonian, the variational ansatz, and the QMC algorithms used to integrate the model, we also discuss an approach to regularize the El-QDO method in the short-range limit to avoid divergences. To regularize the interaction potentials, we classify various damping schemes present in the literature,^11,60,71,82,83^ selecting the most effective one based on systematic tests on argon and water dimers. These systems are employed as prototypes for dispersion interactions and hydrogen bonds, respectively. Since the El-QDO model does not contain the explicit contribution arising from the electronic exchange (that needs to be further developed), here we compare our binding energies with state-of-the-art symmetry-adapted perturbation theory (SAPT) calculations. In particular, by comparing the regularized potential-energy surfaces (PESs) with the SAPT energy contributions, we are able to classify the binding-energy components that are recovered by the improved El-QDO model, opening the way for future construction of the short-range repulsive terms necessary for further generalizations.

The paper is structured as follows: in Section 2 we describe the complete El-QDO Hamiltonian of the mixed system of electrons, nuclei, QDOs, and point charges; in Section 3 we provide details of the implementation of the QMC algorithms for the El-QDO systems; in Section 4 we show in detail our proposed variational ansatz for the El-QDO wave function; in Section 5 we discuss the computational cost and scaling of the El-QDO method; while in Section 6 we provide the computational details of the calculations. In the Results, Section 7, we first study the dissociation curves of El-QDO argon and water dimers categorizing the efficiency of the damping functions used to regularize the description of the short-range interactions, curing overpolarization effects; afterward, we report the study of the solvation energies for the benzene dimer in an environment composed of 50 water molecules, and for the water dimer in a cage of 28 water molecules, as a function of the expansion and contraction of the cages including the zero-temperature equilibrium geometries.^69,70^ Finally, in Section 8 we summarize our findings and anticipate further extensions of the model and possible applications.

## 2 Hamiltonian of Interacting Atoms and QDOs

### 2.1 Quantum Drude Oscillators

The Quantum Drude Oscillator (QDO) model for long-range interactions is constructed from an approximation of the fluctuation–dissipation theorem,^84^ which defines the correlation energy of a system in terms of the charge response function. In the QDO model, the intermolecular interactions, namely polarization and dispersion, arise from the quantum fluctuations of the charge densities and their mutual interactions.

Each QDO consists of a classical center of charge + *q* and a distinguishable quantum particle of charge −*q* and mass μ called *drudon*. The drudon interacts with the center via a quadratic potential
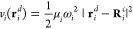
1where **R**_*i*_^*c*^ is the fixed position of the center of the QDO, **r**_*i*_^*d*^ is the position of the quantum drudon and the frequency ω_*i*_ determines the slope of the quadratic well. A system of *N*_*Q*_ QDOs is then fully defined by a set of parameters {**R**_*i*_^*c*^, *q*_*i*_, μ_*i*_, ω_*i*_}_*i* = 1_^*N*_*Q*_^. The fact that each QDO differs in the parameter **R**_*i*_^*c*^ leads to the fact that in this Hamiltonian all drudons are distinguishable particles characterized by a different interacting potential.

In order to add the electrostatics into the model, the static multipolar moments of isolated molecules (e.g., dipole moment of the polar water molecule) have to be included via additional point charges defined by positions, charges, and indices of the parental QDO {**R**_*i*_^*pc*^, *Q*_*i*_, *p*_*i*_}_*i* = 1_^*N*_*pc*_^ for *N*_*pc*_ point charges.

The total Hamiltonian of an interacting system with *N*_*Q*_ QDOs and *N*_*pc*_ point charges has the form
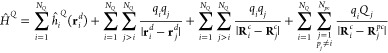
2where *ĥ*_*i*_^*Q*^ are one body operators
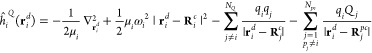
3Each drudon thus interacts via Coulomb potential with all the other charged particles in the system, except for its center, where it feels the quadratic attraction instead, and for the point charges belonging to its parental QDO, where the interaction is omitted.

Using perturbation theory, it is possible to show that the Hamiltonian in eq 2 recovers polarization and dispersion components of the interactions, where the corresponding dispersion coefficients *C*_*i*_ and polarizabilities α_*i*_ depend on the parameters of the QDOs. This allows us to parametrize the QDOs using the leading order response properties of the real system, for example by using the relations ,  and  as discussed in ref (60). The parameters for the argon atom and water molecule considered in this work are reported in Table 1.

**Table 1:** Parameters (in Atomic Units) of the Quantum Drude Oscillators Used in This Work

	*q*	ω	μ
Ar^60^	1.3314	0.7272	0.3020
H_2_O^71^	1.1973	0.6287	0.3656

In order to reproduce the dipole moment of an isolated water molecule, the QDO based model of water,^71^ is built on the TIP4P force field model,^85^ which consists of two point charges on the pseudo-Hydrogen atoms (*q*_H_ = 0.605), and one point charge on the point *M* (*q*_*M*_ = −1.21) placed along the bisector of the angle *∠*HOH at a distance of *R*_OM_ = 0.2667Å from the oxygen atom. Within this model *O* is in practice a ghost atom without any charge that only serves to define the position of the point *M* and of the *H* atoms. The *∠*HOH angles and *R*_OH_ distances in this work are taken from the atomistic geometries of the various cages.

It is important to highlight here that drudons are distinguishable spin-less particles, and thus the short-range repulsion coming from the exchange interactions in electronic systems is missing in the model. This issue is traditionally tackled by introducing a pairwise external repulsion, which is interpolated by using exponential functions^57,60,68^ with the aim of reproducing the interaction potentials of fully electronic systems.

### 2.2 Quantum Embedding of Electrons and Quantum Drude Oscillators

**Figure 1:**
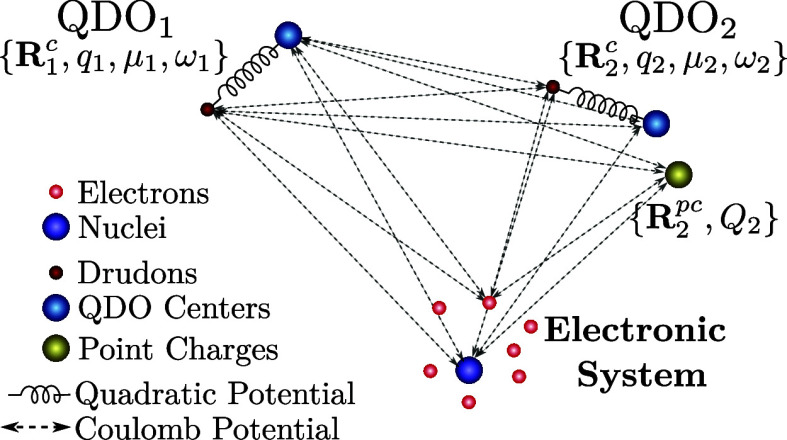
Schematic of the Hamiltonian in eq 4 in the case of an electronic system interacting with two interacting QDOs, where QDO_2_ has one additional point charge (only selected interactions between the QDOs and the electronic system are shown).

A system of QDOs and point charges can be used as a bath for the subsystem of electrons and nuclei, able to reproduce the quantum effects responsible for the long-range interactions between the main fragment and the environment in the real matter. If we consider an electronic system containing *N*_*n*_ nuclei defined by positions and charges {**R**_*i*_^*n*^, *Z*_*i*_}_*i* = 1_^*N*_*n*_^, and *N*_*e*_ electrons, and a bath of *N*_*Q*_ QDOs with parameters {**R**_*i*_^*c*^, *q*_*i*_, μ_*i*_, ω_*i*_}_*i* = 1_^*N*_*Q*_^ and *N*_*pc*_ point charges with parameters {**R**_*i*_^*pc*^, *Q*_*i*_, *p*_*i*_}_*i* = 1_^*N*_*pc*_^ the total El-QDO Hamiltonian will have the form
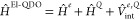
4where the first term is the standard electronic Hamiltonian within the Born–Oppenheimer approximation
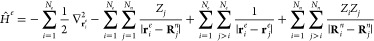
5*Ĥ*^*Q*^ is the Hamiltonian of interacting QDOs and point charges from eq 2 and the last term
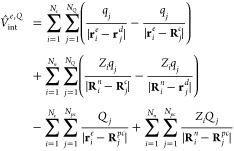
6is the Coulomb interaction between the two subsystems, atoms/molecules, and the QDOs’ with point charges. A schematic of the interactions from the Hamiltonian in eq 4 is shown in Figure 1.

The presence of the correct electrostatics, polarization, and dispersion interactions between the two subsystems described through the El-QDO Hamiltonian in eq 4 was demonstrated in ref (57) on a set of noble gas and water dimers and on small molecules embedded in water environments of various sizes.

Similarly to the pure QDO system discussed before, also the El-QDO Hamiltonian does not include the short-range repulsion coming from the exchange interactions of the full electronic representations. Following the works on pure interacting QDOs,^60,68^ in ref (57) the problem was tackled by fitting the pairwise external repulsion between the QDOs and the electronic subsystem.

### 2.3 Damping of the Coulomb Potential

**Figure 2:**
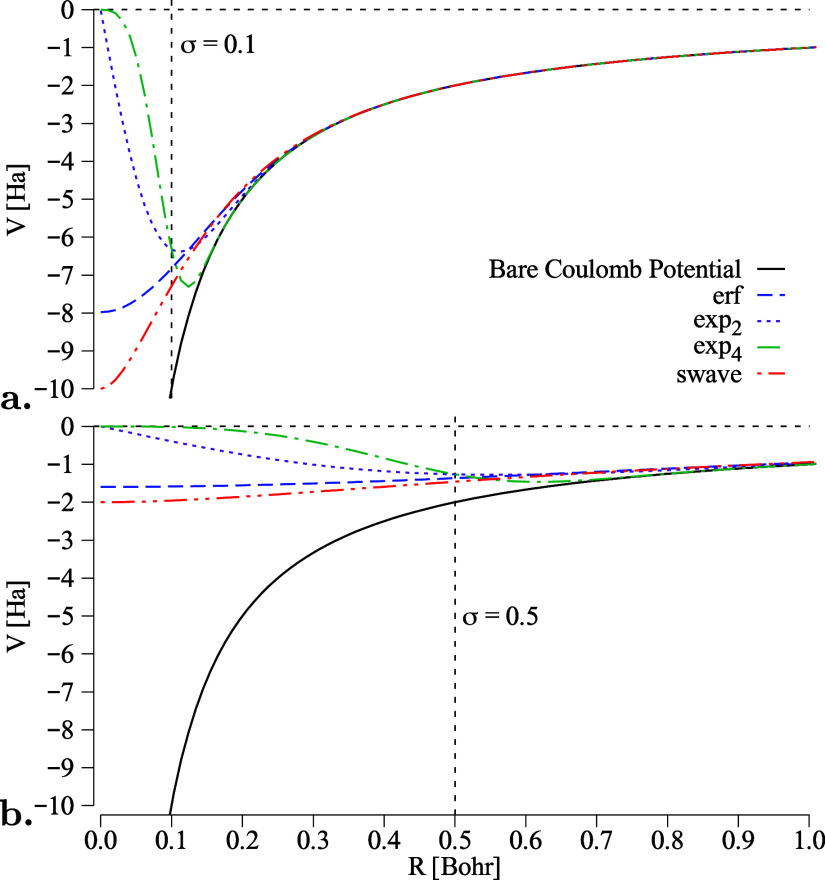
Damped Coulomb potentials from eqs 7–10 compared to bare Coulomb for σ = 0.1 Bohr and σ = 0.5 Bohr. The values of σ are indicated via the dashed vertical lines.

In practice, the use of bare Coulomb potential for the interactions between the electrons and the QDOs’ environment and between interacting QDOs displays nonphysical behavior in the short-range region. This is an effect well-known from QM/MM methods,^82^ often referred to as polarization catastrophe or charge spilling. To minimize this issue, damping functions are introduced for these interactions, changing the shape of the Coulomb potential in the short-range region. In this work, we explore the effect of four functional forms of the damping functions from the available QM/MM and QDO literature, namely the error function (erf)^71^
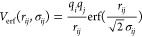
7the Gaussian damping (exp_2_)^11^
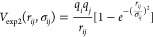
8the exponential with fourth power (exp_4_)^60^
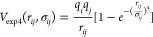
9and the s-wave expansion (swave)^82,83^
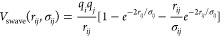
10where σ_*ij*_ is specified for all pairs of particles using the combination rule . Examples of the four damping functions, compared to the bare Coulomb potentials are shown in Figure 2 for values of the damping parameter σ = 0.1 Bohr and σ = 0.5 Bohr.

Clearly, these expressions of the damping functions are not exclusive and can be substituted also with simpler polynomial expansions that have a lower computational cost, such as the one proposed by Pathak and Wagner in ref (86) to regularize the forces in quantum Monte Carlo.

## 3 Quantum Monte Carlo Methods

Quantum Monte Carlo (QMC) methods are a family of stochastic techniques for integrating the many-body time-independent Schrödinger equation over a chosen trial wave function.^78−80^

In this work, we discuss in detail the implementation of two of these methods, namely the variational Monte Calo (VMC) and diffusion Monte Carlo (DMC),^78−80^ with a focus on the adjustments needed in order to integrate simultaneously the drudonic and electronic degrees of freedom, as first done in ref (57). These methods have been implemented in QMeCha α.0.3.0,^87^ a QMC package published privately on GitHub.

In the rest of this section, we will indicate with **r̅** the vector of the coordinates of all the quantum particles in the system, both electronic and drudonic, and only when necessary we will explicitly introduce the labels for electrons or drudons.

### 3.1 Variational Monte Carlo and Wave Function Optimization Methods

Variational Monte Carlo^78−80^ is based on the stochastic integration of the energy functional of a given Hamiltonian *Ĥ* over the chosen many-body variational ansatz Ψ_*T*_(**r̅**)
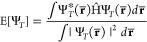
11which is done by separating the integrand into a product of two functions
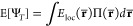
12that are respectively the probability density  of finding the system in a configuration **r̅** and the local energy , that is the energy of the system in that particular configuration. The integration in eq 12 is obtained through a random walk (or an arbitrary number of parallel random walks) evolved according to the Metropolis-Hastings algorithm.^88,89^ By computing the local quantities, such as the energy *E*_loc_(**r̅**), for a sequence of  uncorrelated configurations, it is possible to obtain a stochastic estimation of the total energy through the mean value of the corresponding local quantities accumulated over the entire evolution
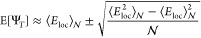
13with an error that decreases as the  depending on the variance .

The extension of the VMC algorithm to integrate a mixed system of drudons and electrons is rather straightforward. In our approach, the two sets of particles are diffused particle-by-particle in random order starting from the electrons, according to the Metropolis-Hastings algorithm.^88,89^ Each particle’s trial move is proposed according to
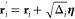
14where **η** is a 3-dimensional vector of Gaussian distributed random numbers with zero mean and unitary variance, and Δ_*i*_ is an amplitude that depends on the type of particle and is defined as
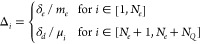
15The parameters δ_*e*_ and δ_*d*_ are two amplitudes used respectively for the electrons and for the drudons, and *m*_*e*_ and μ_*i*_ are respectively the mass of the electrons (which is equal to 1) and the mass of the *i*th drudon. The amplitudes δ_*e*_ and δ_*d*_ are optimized at the beginning of the MC run by converging the acceptance probability of the moves to the value of 50%, which is the rule-of-thumb that balances the acceptance rate of the single particle moves and the autocorrelation between configurations and thus observable values. This procedure is repeated until  configurations are sampled.

Within this VMC scheme, it is also possible to optimize the trial wave function through energy (or variance) minimization.^90−98^ In this work, the set of parameters is optimized through the Stochastic Reconfiguration procedure described in refs (95, 99) with the use of correlated sampling technique^100^ in order to better estimate the energy variation in between parameter updates. The wave function’s sampling is automatically recomputed if the overlap between two consecutive wave functions becomes lower than a chosen threshold.

### 3.2 Diffusion Monte Carlo

As previously discussed, for the VMC method the quality of the description of the ground state and its energy strictly depends on the parametrization of the trial wave function Ψ_*T*_(**r̅**), and so on the dimension of the variational space.^78−80^ To obtain a more accurate estimation of the physical observable, better describing the dynamical correlation between the particles in the system, it is common to use the DMC method within the Fixed-Node (FN) approximation^101^ required to overcome the sign problem that arises when dealing with Fermionic systems.^102,103^

Here we avoid to recall the general description of the FN-DMC algorithm, and we want only to focus on the extensions that have been made to integrate both the electronic and drudonic degrees of freedom.

The first change is related to the drift/diffusion process at finite time step δτ, in which the particles’ positions are updated with a particle-by-particle scheme, and the evolution from time interval *m* to *m* + 1 is written as
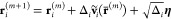
16where **η** is a 3-dimensional vector of random variables extracted with a Gaussian distribution with zero mean value and unitary variance, and  is the drift velocity  rescaled according to the procedure introduced by Umrigar et al.^104^ to avoid divergences near the nodal surface, and Δ_*i*_ are the rescaled single particle time steps defined, similarly to what has been done in VMC, as
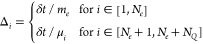
17

During the reweighting phase, to avoid numerical instabilities near the nodal surface of the trial wave function for finite time steps δτ, we use the cutoff  introduced by Zen et al. in ref (105), where α is a tunable parameter, here set to 0.2.

Finally, in this work, to treat the atoms of the electronic systems we use pseudopotentials in order to reduce the computational cost of the calculations. Within the FN-DMC algorithm, the integration of the nonlocal part is usually treated through locality approximation^106^ or T-Move.^107^ Here, to reduce the possible dependency of the results on the Jastrow factor, we apply determinant locality approximation (DLA)^108^ in which the nonlocal operator is evaluated using only the Slater determinant part of the many-body wave function.

## 4 Wave Functions for Interacting Electrons and Drudons

The variational ansatz used to approximate the ground state of the El-QDO Hamiltonian in eq 4 depends explicitly on the 3*N*_*e*_ electronic **r̅**^*e*^ and 3*N*_*Q*_ drudonic **r̅**^*d*^ coordinates, while the coordinates of the nuclei **R̅**^*n*^ and of the QDOs’ centers **R̅**^*c*^ are considered as fixed parameters within the Born–Oppenheimer approximation. As first proposed in ref (57), the full wave function is constructed as the product of three independent terms
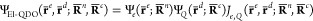
18which correspond to the electronic wave function Ψ_*e*_(**r̅**^*e*^;**R̅**^*n*^), the pure QDO wave function Ψ_*Q*_(**r̅**^*d*^;**R̅**^*c*^) and a positive-definite coupling function *J*_*e*,*Q*_(**r̅**^*e*^,**r̅**^*d*^;**R̅**^*n*^,**R̅**^*c*^) that describes the correlation effects between the electronic subsystem and drudonic environment.

The ansatz in eq 18 does not depend explicitly on the positions of the point charges that represent only an external potential, that polarizes the electrons and the drudons of the other QDOs. For pure electronic or pure QDO systems only the corresponding Ψ_*e*_(**r̅**^*e*^;**R̅**^*n*^) or Ψ_*Q*_(**r̅**^*d*^;**R̅**^*c*^) are used.

### 4.1 Electronic Wave Function

The electronic part Ψ_*e*_, which depends only on the electronic coordinates and on the nuclear positions as parameters, is written as the product
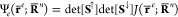
19of the Slater determinants for the spin up and spin down electrons, where **S**^*↑*^ and **S**^*↓*^ are respectively the Slater matrices of the molecular orbitals occupied by the two spin populations, and a Jastrow factor *J*(**r̅**^*e*^;**R̅**^*n*^).^109^ The molecular orbitals that define the elements of the Slater matrix **S**(**r̅**^*e*^) are written as linear combinations
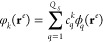
20of *Q*_*S*_ contracted Gaussian type orbitals ϕ_*q*_(**r**) centered only on the nuclei **R̅**^*n*^ of the electronic system and not on the QDO centers. The bosonic Jastrow factor^109^ used in this work is inspired by the one introduced by Casula et al. in ref (110), as a sum of two terms
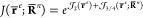
21which can be classified as a pure homogeneous two-body term  and a three/four body inhomogeneous term  that is used to describe the Fermionic pair correlations in the field of the nuclei.

The homogeneous two-body Jastrow factor is written as the sum of functions depending only on the distances between electron pairs *r*_*ij*_ = |**r**_*i*_^*e*^ – **r**_*j*_^*e*^|
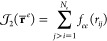
22where the pair correlation functions are written as
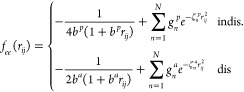
23respectively for indistinguishable (parallel spin) electrons and distinguishable (antiparallel spin) ones. The variational parameters *b*^*p*^ and *b*^*a*^ are related to the cusp functions and are optimized independently.^111^ The additional linear combination of Gaussian terms works as a remodulating factor that depends on the set of coefficients *g*_*n*_^*p*^ and *g*_*n*_^*a*^ and exponents ζ_*n*_^*p*^ and ζ_*n*_^*a*^ that are also optimized.

The nonhomogeneous three/four body Jastrow term is written as the linear combination of products of two atomic orbitals
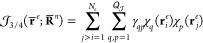
24where  is the total number of non-normalized atomic orbitals χ_*q*_(**r**^*e*^) in the Jastrow basis set, and the parameters γ_*qp*_ describe the correlation of two electrons in the field of the nuclei. Since the Jastrow factor must be symmetric with respect to the exchange of all the electrons, the γ_*qp*_ parameters satisfy the condition γ_*qp*_ = γ_*pq*_.

The computational cost of evaluating this Jastrow term can be understood considering that there are *N*_*e*_(*N*_*e*_ + 1)/2 terms, requiring vector-matrix-vector multiplications that have leading computational cost of . Assuming *Q*_*J*_ ∼ *N*_*e*_, the naive initial computation of this Jastrow factor will be of *O*(*N*_*e*_^4^). Each Jastrow update for a displacement of one single electron during the random walk would cost *O*(*N*_*e*_^3^). In truth, taking advantage of the fact that the Jastrow does not explicitly depend on the distances between electronic pairs, and the coupling coefficients γ_*qp*_ are identical for all electronic pairs, it is possible to reduce the computational cost to a series of vector sums, and only *O*(*N*_*e*_^2^) multiplications.

### 4.2 Drudonic Wave Function

The QDO part Ψ_*Q*_(**r̅**^*d*^;**R̅**^*c*^) of the total El-QDO wave function depends only on the coordinates of the drudons and parametrically on the centers of QDOs. Its functional form resembles that of the exact solution of a system of QDOs interacting via dipole potentials.^59,60^ It is written as the exponential
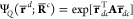
25of the vector-matrix-vector product between the vector **r̅**_*dc*_ = **r̅**^*d*^ – **R̅**^*c*^ of the 3*N*_*Q*_ components of the displacements of each drudon from its center, and the square symmetric matrix **A** containing 3*N*_*Q*_(3*N*_*Q*_ + 1)/2 independent parameters that explicitly correlate fluctuations of the different QDOs. Despite being just an approximation of the exact QDO wave function, ansatz in eq 25 does not introduce a bias into the final DMC energies, because, due to the distinguishability of the drudons, it does not contain a nodal surface.

### 4.3 Electron-Drudon Coupling

The last part of the total wave function is the coupling *J*_*e*,*Q*_(**r̅**^*e*^, **r̅**^*d*^;**R̅**^*n*^, **R̅**^*c*^) between two types of quantum particles in the El-QDO system. We can again suppose that the Coulomb potential between the electronic system and the QDO environment is well approximated by dipole potential at large separations, so even in the case of full Coulomb interactions we can approximate the wave function using the form^57^
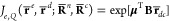
26where **r̅***_dc_* is the vector of the displacements of each drudon from its center, defined in the previous section, while **μ** is the 3-dimensional vector of the dipole moment of the electronic subsystem, depending only on the configuration of the electrons **r̅**^*e*^
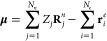
27and **B** is a matrix containing 3 × 3*N*_*Q*_ free parameters.

In the limit in which the electronic subsystem and the drudonic environment only interact through noncovalent bonds, the term described in eq 26 has the purpose of recovering the dynamical correlation between the electrons and drudons. This factor is chosen to be always positive, since it recovers correlation that is responsible for dispersion, polarization, and electrostatic effects that change the nodal structure of the electronic subsystem only indirectly, through the deformation of the electronic part. Furthermore, it has been shown that the effects of the environment on the electronic subsystem have very small effects on its nodal structure.^57,112^

For this reason this dipolar approximation for the *J*_*e*,*Q*_(**r̅**^*e*^, **r̅**^*d*^;**R̅**^*n*^, **R̅**^*c*^) coupling is sufficiently accurate when used in combination with DMC, as done in this work.

## 5 Computational Cost of the El-QDO Method

One of the advantages of the El-QDO approach resides in its efficiency with respect to other quantum embeddings that are able to include explicit dynamical many-body correlation effects between the environment and the electronic subsystem. In QMC methods, the computational cost of the stochastic integration process is usually proportional to *N*_*p*_^3–4^ being *N*_*p*_ the number of particles in the system, where the difference in the exponential factor depends on the complexity of the wave function. This relationship holds for Fermionic particles, yet for the QDOs in the environment, the overall computational problem becomes simpler. Since each QDO has a single quantum particle, i.e., a drudon, that is intrinsically distinguishable from all other particles in the Hamiltonian^59^ (drudons and electrons), the total QDO wave function reduces to the simple dipole wave function or at most to a Slater product of single-particle orbitals correlated only through a two-body Jastrow factor.^59^ This simple mathematical representation of the QDO environment leads to a reduction of the scaling cost with respect to the number of drudonic particles, which is essentially quadratic, i.e., proportional to the product between the number of particles and the dimension of the basis set used to construct the wave function. The lower scaling of the QDO environment with respect to the electronic subsystem as a function of the number of particles leads to the advantage that the computational cost of the environment becomes nearly negligible^57^ with respect to the computational cost of integrating the electronic subsystem. For example for the benzene dimer (60 electrons when substituting the core electrons of the carbon atoms with pseudopotentials), 1000 individual DMC steps without the water cage take 29.7 s, while the time for the same number of steps with the addition of 50 QDOs (50 drudons) increases only to 31.9 s, and this is a trend that can be seen overall in the calculations. In practice, the environment reaches the same computational cost as the Fermionic subsystem when its number of particles is proportional to the square of the number of particles of the subsystem.

Naturally, this advantage goes beyond the El-QDO method in the framework of QMC. In fact, the generalization of the El-QDO approach in other ab initio frameworks such as Configuration Interaction or Coupled Cluster is trivial and would also benefit from the low-scaling of the QDO environment with respect to the number of distinguishable drudonic particles.

This generalization can be better understood by separating the point charges and the QDOs in the environment. The interaction between the electronic subsystem and the point charges in the environment resembles the one that appears in QM/MM approaches, and its construction can follow the same procedures. Regarding the QDO part, the interaction with the electronic subsystems can be easily modeled in wave function based methods, such as HF, CC, or CI through the construction of a total wave function, built as the sum of products of an electronic Slater determinant and a drudonic Slater product. Similar considerations hold for DFT, where to integrate the electronic subsystem and the drudonic one, it could be possible to follow the same procedures used for QM/QM approaches, such as those used in DFT/CC or DFT/CI embeddings.^113^

## 6 Computational Details

The SAPT calculations have been performed in PSI4 package,^114^ and the basis set specifications are discussed in the results section for each system considered.

The molecular orbitals used to describe the determinant part of the total wave functions, used in QMC calculations, were obtained from DFT calculations using the PBE0 functional^115^ with the GAMESS (2016 R1)^116^ or ORCA 5.0^117^ software. All these calculations have been done using ccECP effective core potentials^118−121^ with the corresponding cc-pVDZ Gaussian basis sets (GTOs) for the argon dimers calculations, the aug-cc-pVDZ basis for water dimers, and the aug-cc-pVTZ for all the calculations of benzene and water dimers in the QDO water cages and in vacuum. The dynamical Jastrow factor is constructed from 3s2p1d uncontracted GTOs for all the heavy atoms and from 2s1p uncontracted GTOs for the Hydrogen atoms. The Slater determinant of the electronic part was fixed during the wave function optimizations at the VMC level of theory. A time discretization step of *d*τ = 0.005 au has been used for all FN-DMC calculations.

## 7 Results and Discussion

In this work, we advance the results presented in ref (57) by concentrating on generalizing the El-QDO procedure also to equilibrium geometries and short-range regions dominated by repulsive exchange effects. As discussed in the introduction, these regions were not investigated in the previous work^57^ due to overpolarization effects^11,60,71,82,83^ that lead to large uncertainties in the estimation of the interaction energies. In QMC, during the dynamical sampling, these effects can cause the drift of the electrons toward the positive charges of the QDOs or toward the positive point charges added to model implicit molecular dipoles in the environment.

To overcome these challenges, in this work we first study the different damping schemes present in the literature (summarized in Section 2.3) to cure artificial overpolarization effects in QM/MM methods.^11,60,71,82,83^

The efficiency of the damping functions is verified and compared by studying the potential energy surfaces (PESs) of the argon and water dimers, which are prototype systems for van der Waals and hydrogen bonded systems, modeled via the El-QDO approach.

Finally, using the most efficient of these regularizing functions, we study the solvation energies of two molecular dimers, the benzene and the water dimers, described at the electronic level, embedded in cages of QDO-modeled water molecules. By studying the solvation energies as a function of the artificial compression and expansion of the cages, comparing the results with the energy decomposition given by symmetry-adapted perturbation theory (SAPT), we are able to qualitatively identify and classify the energy components that are captured by the El-QDO model.

### 7.1 Argon Dimer

The interaction energy of the argon dimer is dominated by attractive dispersion in the long-range limit and by exchange repulsion in the short-range one, making it a prototype for van der Waals systems and an important benchmark for computational methods.

As shown in previous works,^57,59,64^ both the model of two Coulomb interacting QDOs and the mixed El-QDO system, in which one atom is modeled by a QDO while the other is described through the many-electron Hamiltonian, are able to correctly describe the dispersion energy of the argon dimer in the long-range limit, in which it corresponds essentially to the total interacting energy.^57,59,64^

**Figure 3:**
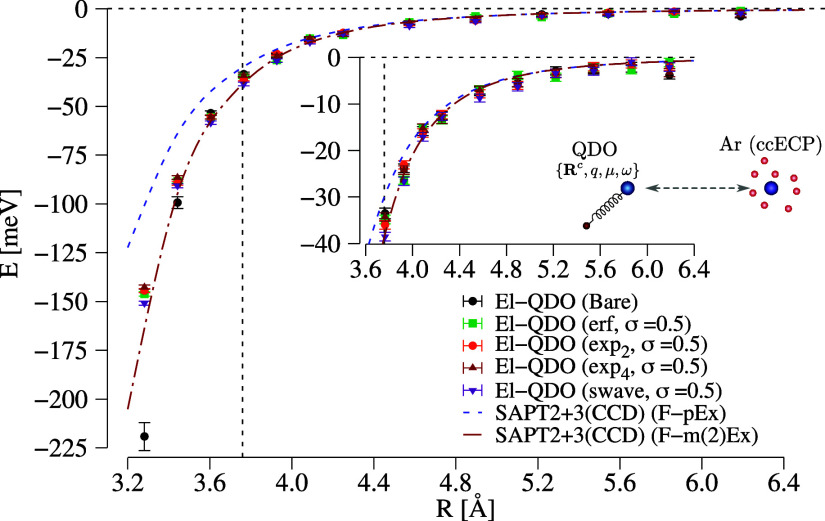
Interaction energies of the El-QDO argon dimer for erf, exp_2_, exp_4_, and swave damping functions as a function of the distance between the two subsystems. The results are compared to two SAPT2 + 3(CCD) curves explained in the main text. The vertical dashed line is the equilibrium geometry of the argon dimer.

This same conclusion can be obtained by observing the panels in Figure 3 where the El-QDO PESs obtained with different damping functions are compared to PESs obtained from two subsets of energy contributions computed with SAPT2 + 3(CCD) calculations with the aug-cc-pVQZ basis set.^122−124^ The F-pEx curve is obtained by removing from the total SAPT energy all the pure exchange contributions, while the F-m(2)Ex is obtained by removing also the mixed terms which include exchange up to only the second order. The reason behind the construction of the F-m(2)Ex PES comes from the fact that the energy contributions of the individual terms in the full expansion do not converge monotonically with increasing order. For example, the Exchange-Induction(30)^125^ term is larger than the corresponding two lower-order terms, and in the total interaction energy is compensated by the Induction(30) term.^125^

In the long-range limit, all the curves are in agreement, and around the equilibrium geometry, only small discrepancies start appearing between the El-QDO, the F-pEx, and the F-m(2)Ex PESs, clearly due to the short-range exchange contributions.

Yet, as the atoms come near each other, the El-QDO curve without regularization (Figure 3) tends to deviate from the others diverging to negative values, together with the variance of the energy estimator. This effect is due to the overpolarization that during the DMC sampling drifts the electrons toward the QDO center. It follows that through the use of the regularizing damping functions, in Figure 3 we show the results with σ = 0.5 Bohr (The El-QDO dissociation curves for various values of the damping parameter σ can be found in the Supporting Information in Figures S2 and S3), the divergence is always cured and the El-QDO PESs converge in between the F-pEx and the F-m(2)Ex curves which vary from one another in the ‘repulsive’ region of less than 50 meV.

Overall, we can conclude that for this particular system all the types of damping functions give consistent results, and can be used to regularize the short-range interactions in the El-QDO approach. Clearly, this regularization is a fundamental step to describe the repulsive short-range exchange necessary to reproduce the full PES.

### 7.2 Water Dimer

The second system studied in this work is the water dimer, a prototypical dimer for hydrogen bonding. Here we present the potential energy surface computed for the most stable conformer fixing the structures of the monomers along the dissociation path. In the case of the El-QDO calculations, the QDO represents the donor monomer (accepting an electron) while the acceptor is described through the electronic Hamiltonian.

**Figure 4:**
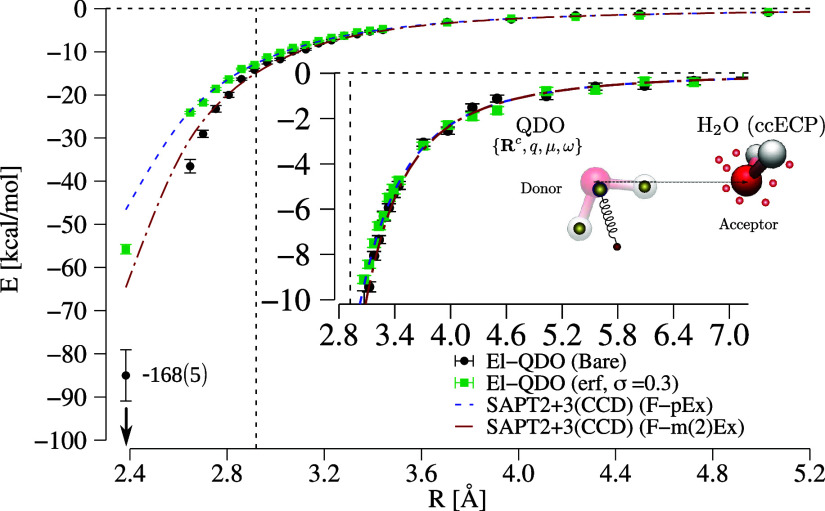
Interaction energies of the El-QDO water dimer for the error function damping as a function of the distance between the two subsystems. The results are compared to two SAPT2 + 3(CCD) curves explained in the main text. The vertical dashed line is the equilibrium geometry of the water dimer.

As done for the argon dimer, in Figure 4 we compare the El-QDO interaction energies, obtained with the error function damping with σ = 0.3 Bohr (El-QDO curves for the other three damping functions and various values of the damping parameter σ can be found in Supporting Information in Figures S4 and S5), to the same two F-pEx and the F-m(2)Ex PESs constructed from the different sets of contributions obtained from SAPT2 + 3(CCD) calculations using the aug-cc-pVQZ basis set.^122−124^

Since the water molecule has an intrinsic dipole and the interaction in the dimer has strong charge transfer contributions the overpolarization effect of the El-QDO model with the bare Coulomb interactions is even larger than that observed for the argon dimer. Yet, again, as for the previous dimer, also for this system the tuning of the damping parameter leads to the convergence of the El-QDO PES in between the F-pEx and F-m(2)Ex curves discussed above.

This result strengthens what was previously observed for the noble gas dimer. The regularizing damping functions are efficient in taming overpolarization, and their effect must be balanced when constructing the additional repulsive term responsible for the explicit description of exchange effects.

### 7.3 Benzene Dimer Embedded in 50 Water Molecules

To test the El-QDO model on more challenging systems we consider the benzene dimer embedded in a first shell of 50 water molecules obtained as a snapshot in molecular dynamics simulations.^126^

This system was first used as a test case for the El-QDO method in ref (57). In that previous work, the water cage was expanded to avoid the overpolarization error so that the minimum distance between the water molecules and the benzene dimer was larger than 3.4 Å (the cage was artificially spread by d*R* = 1.5 Å). For that level of cage stretching, the interactions between the electrons of the dimer and the QDOs of the environment remain dominated by the dispersion contribution. At the same time, polarization remains weak, removing any possible problematic behavior that could appear in the short-range. The contraction and expansion of the cage is obtained by first evaluating the geometric center of the nuclei of the electronic subsystem. From this reference center we define the vector of the positions of the Oxygen atoms **R**_*O*_ that is modified for a scalar value dR according to the equation . The Hydrogen pairs are translated according to their Oxygen atom in such a way that the relative angles are preserved and the intermolecular structures remain fixed. Clearly this can become problematic when the cage is severely contracted, but in this work this procedure is sufficient as a means to explore the short-range behavior of the El-QDO model.

**Figure 5:**
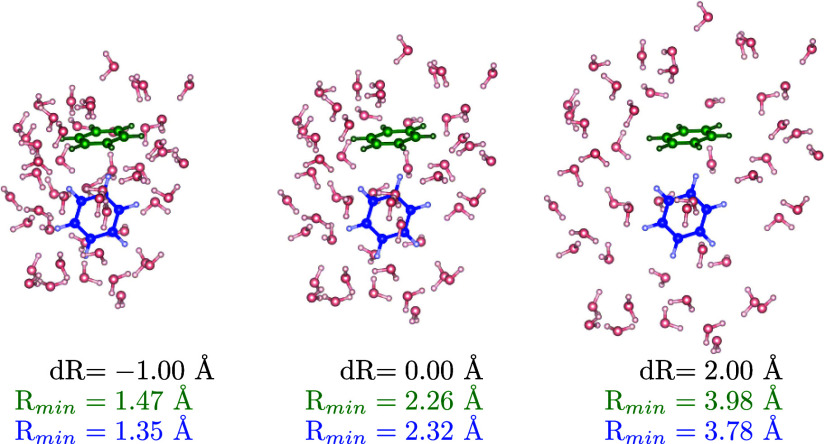
Monomer_1_ (green) and monomer_2_ (blue) in the environment composed of 50 water molecules for three values of the expansion of the water cage from its center. *R*_min_ are the minimal atom–atom distances between the monomer of the corresponding color and the environment. The original geometry for d*R* = 0.0 Å is taken from ref (126).

Here, by introducing the regularization of the potential through the error function with σ = 0.5 Bohr, we extend the study of the system as a function of the expansion and compression of the cage including the ab initio zero-temperature equilibrium geometry (d*R* = 0.0 Å in Figure 5).

In Figure 5 we can see, together with the equilibrium geometry (central plot), the molecular system for the maximum compression (d*R* = −1.0 Å) and maximum expansion (d*R* = 2.0 Å) for which we also report the maximum and minimum values of the atomic distances.

**Figure 6:**
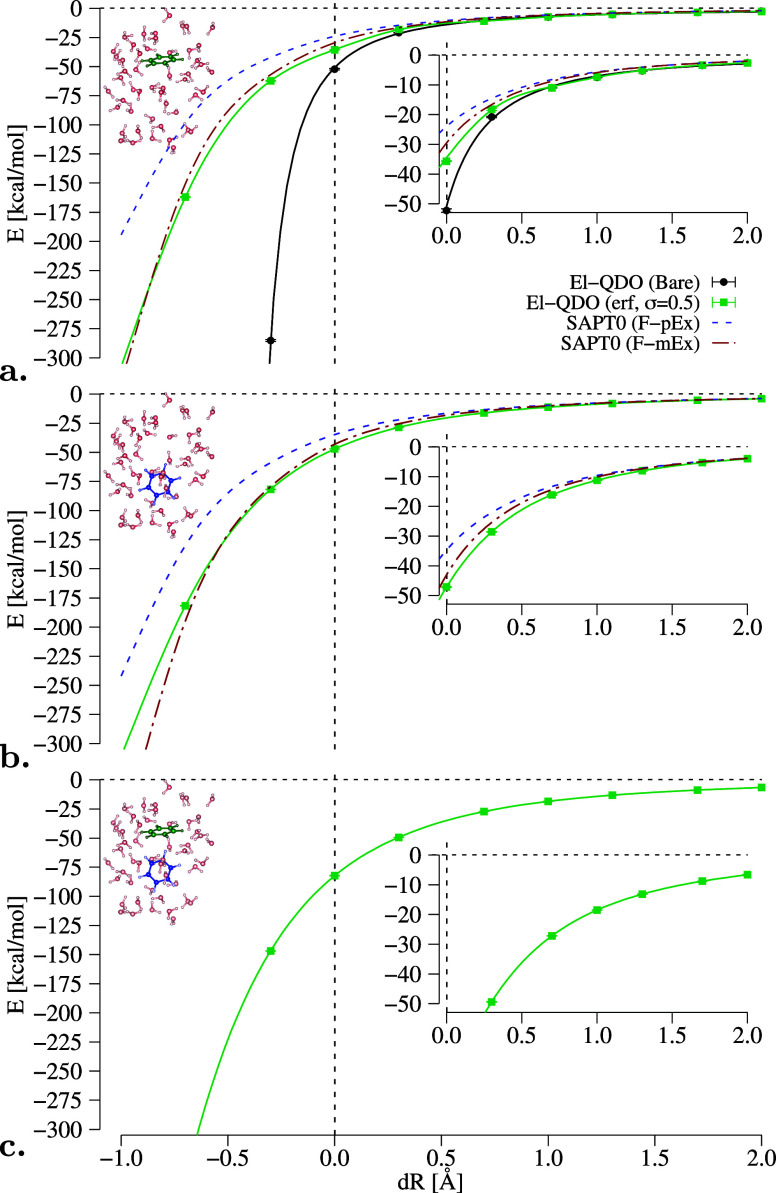
Solvation energies of benzene (monomer_1_, monomer_2_, and dimer) in an environment composed of 50 water molecules as a function of the expansion of the water cage from its center. El-QDO results are compared to the SAPT0 decomposition explained in the main text.

For a set of intermediate points in the interval d*R* ∈ {−1.0:2.0} Å, as done in ref (57), we compute the variation of the solvation energies of the two separate monomers (obtained by alternatively removing one of the two benzene molecules) and of the dimer as a function of the cage deformation, shown in Figure 6.

In the upper panel of the figure, for monomer_1_ we can see that the El-QDO PES with bare Coulomb potential displays overpolarization effects also at the equilibrium geometry. The introduction of the regularizing damping function for the interaction potentials with σ = 0.5 Bohr (other values of the damping parameters for the erf damping function in the case of monomer_1_ can be found in SM in Figures S6 and S7) has the effect of removing the overpolarization error, as previously observed, converging the solvation energy for all values of dR.

We can also notice that the solvation energy curves obtained with the El-QDO method follow the F-mEx and F-pEx profiles obtained with SAPT0 and jun-cc-pVDZ basis set.^127^ Here the accuracy of the SAPT calculation was lowered, with respect to that of the isolated argon and water dimers, due to the computational cost of these large molecular systems.

### 7.4 Water Dimer Embedded in 28 Water Molecules

**Figure 7:**
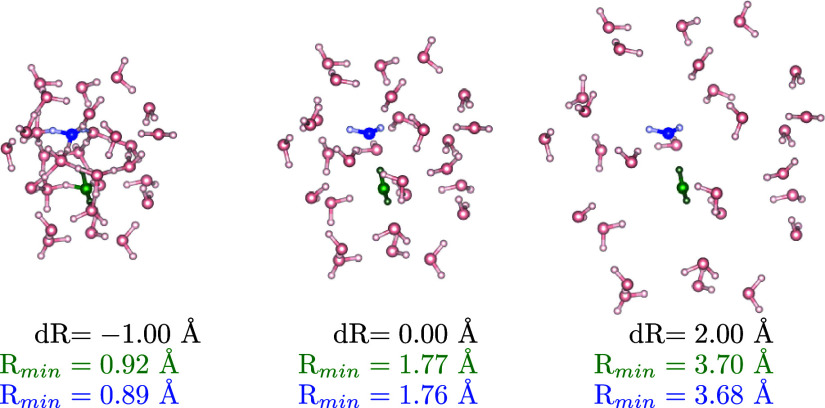
Monomer_1_ (green) and monomer_2_ (blue) in the environment composed of 28 water molecules for three values of the expansion of the water cage from its center. *R*_min_ are the minimal atom–atom distances between the monomer of the corresponding color and the environment. The original geometry for d*R* = 0.0 Å is taken from ref (128).

In the previous system, the overpolarization effects are removed through the use of the regularization function. In order to study a more complex system, in which partial charge transfer is crucial, here we consider a cluster of 30 water molecules in equilibrium at zero-temperature^128^ that is represented in the central panel in Figure 7. Within this system, the water molecules form a network of hydrogen bonds in which all molecules play both the roles of acceptors and donors.

**Figure 8:**
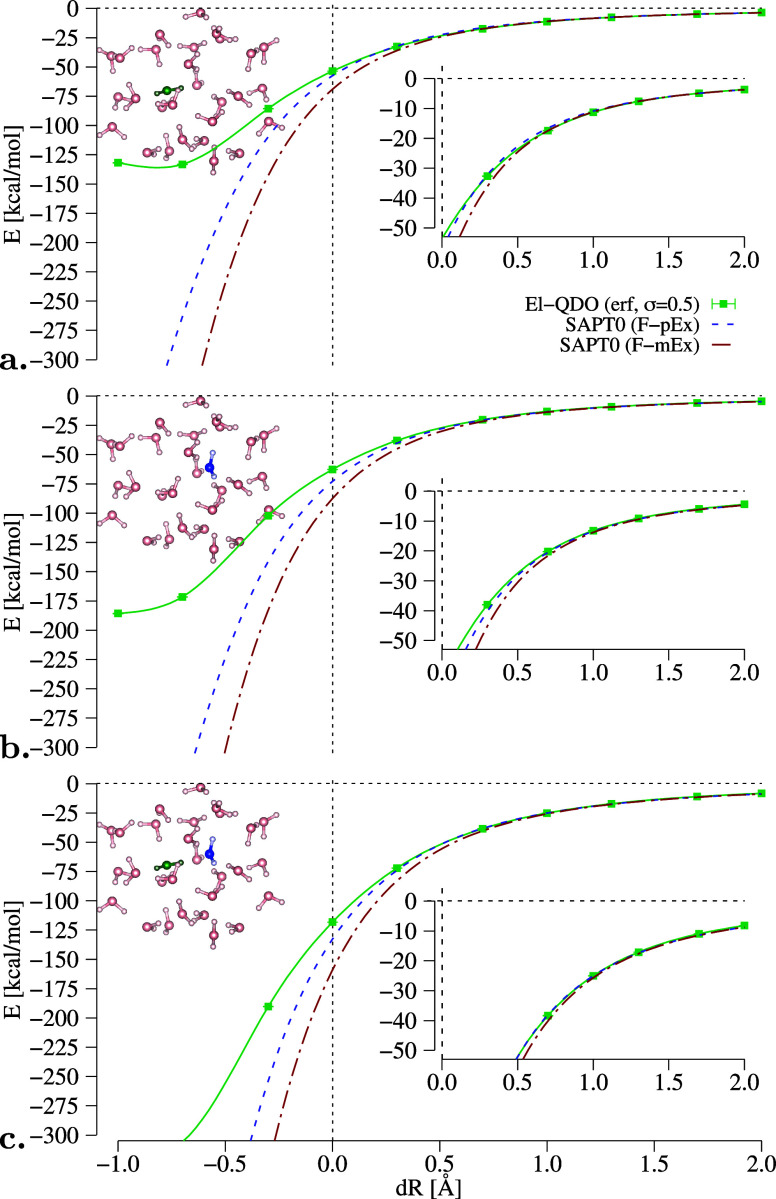
Solvation energies of water dimer and its monomers in an environment composed of 28 water molecules as a function of the expansion of the water cage from its center. El-QDO results are compared to the SAPT0 decomposition explained in the main text.

In the El-QDO approach, the electronic subsystem is represented by the central water dimer, in which the monomers are colored in blue and green in Figure 7. As for the previous benzene system, also in this case we study the solvation energies as a function of the compression and expansion of the cage between the values of d*R* ∈ [−1.0:2.0] Å (Figure 8).

Also, in this case, we can see a substantial qualitative agreement with the two SAPT0 curves obtained with jun-cc-pVDZ basis set^127^ where we have removed the contributions coming from exchange, by using the same damping function used for the benzene dimer in Section 7.3. The discrepancy in the short-range can be explained by considering that while in this system the maximally squeezed cage (d*R* = −1.0 Å) has a minimum distance of only 0.89 Å between the atoms, for the benzene dimer it was much larger, of about 1.35 Å (as can be seen in Figure 5).

Thus, the discrepancy between SAPT0 and El-QDO in the short-range for this system depends on the influence of the regularization function in the potential. We note that in the water dimer in vacuum, discussed in Section 7.2, the minimum distance studied between the hydrogen atom of the donor molecule and the oxygen of the acceptor, was also much larger if compared to this case, around 1.4 Å.

From these results, we can also focus on another interesting aspect of water clusters, that regards the study of the change of the binding energy between the central water molecules that form the electronic water dimer, as a function of the deformation of the cage. Notice in Figure 7 that the structure of the dimer in the center of the cluster is not deformed together with the rest of the cage, thus the change of the binding energy only depends on the change of the polarization effects induced by the modification of the environment.

While in ref (57) for the benzene dimer it was shown that the presence of the environment of QDOs did not substantially affect the binding energy between the two molecules, here the situation appears to be quite different.

**Figure 9:**
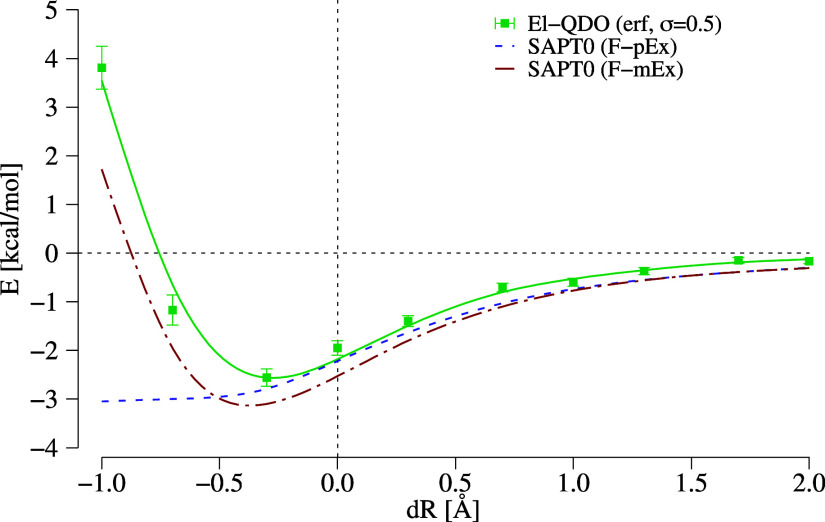
Change of the binding energy (eq 28) of the central water dimer due to the presence of the environment (Figure 7) as a function of the QDOs cage deformation. El-QDO results are compared to the SAPT0 decomposition explained in the main text.

From the separate solvation energies for the single monomers *E*_solv_(M_1_) and *E*_solv_(M_2_), and of the dimer *E*_solv_(D) in Figure 8, we can define the change of the binding energy between the two monomers as
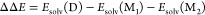
28plotted as a function of the cages’s structural deformation in Figure 9.

For d*R* ≥ 0.0 Å we can identify a substantial quantitative agreement between the SAPT0 results and the El-QDO model, as seen also for the solvation energies of the single components. Interestingly for large compressions d*R* < −0.25 Å the energy profile of the model follows the F-mEx SAPT0 energy profile (while the F-mEx converges to a constant), which strengthens the hypothesis that, despite the effects induced by the regularization, the El-QDO model should be compared to the total SAPT interaction energy minus all the pure and mixed terms which include exchange.

This, on the other hand, is a clear step toward better identifying the required contributions that need to be added to the El-QDO model in the short-range to reproduce full interaction energy profiles.

## 8 Conclusions

In this work, we have advanced the applicability of the El-QDO model first introduced in ref (57), presenting a systematic study of the interactions between electronic systems and QDOs as a means of constructing an embedding approach where the QDOs represent the environment and its dynamic response.

We have detailed the total Hamiltonian defining the interactions between the electrons and the drudons, and we have described the trial wave function used to approximate the ground state of the full quantum system. Afterward, we have outlined the QMC algorithms used to integrate the energy functional with respect to both electronic and drudonic degrees of freedom and to optimize the variational parameters of the wave function.

We then investigated various regularization (damping functions) to stabilize the embedding procedure in the short-range region, improving on the results previously published in ref (57) that were limited to out of equilibrium expanded cages to avoid overpolarization errors and instabilities. To assess their effectiveness in mitigating these errors, also observed in other QM/MM embedding methods, we benchmarked various functions proposed in the literature by reconstructing the PESs of the argon and water dimers using the El-QDO method.

Through these first results, we have found that all damping functions are able to stabilize the El-QDO method at the structural equilibrium, and in the short-range region where repulsion contributions become dominant, for a reasonable choice of the cutoff length σ. For the sake of simplicity, by selecting the error function damping, which is also the most commonly used function in QM/MM, we have employed the El-QDO model to investigate the solvation energies of the benzene and water dimers within cages of 50 and 28 water molecules, respectively, and the variation of the bond energy of the water dimer in 28 water molecules.

These calculations were performed using cages with equilibrium and nonequilibrium geometries, obtained by expanding and contracting the cages.

The comparison of the El-QDO energies with those obtained with different SAPT energy components (with exchange terms selectively removed, i.e., F-pEx and F-mEx sets), has revealed the ability of the regularized El-QDO model to accurately describe the electrostatic, polarization, and dispersion interactions between the electronic subsystem and the environment: quantitatively at long-range to equilibrium distance, and qualitatively at subequilibrium distances.

Naturally, while the accurate description of short-range repulsive terms arising from electronic exchange still remains a challenge and will be the focus of future works, the stabilization of the El-QDO procedure across all distances represents a significant advancement toward the generalization of the method. In the future, it will be possible to extend the El-QDO model to all applications for which the full Coulomb QDO has already been parametrized, such as the NH_3_, CH_4_, and BH_3_ molecules,^60^ or atomic solids or liquids.^61,64^ Clearly, further extensions of the QDO model itself will be required to tackle more complex systems. First of all, the model could be generalized to describe strongly anisotropic systems following the approach proposed in ref (129) or by using one QDO per atom as done in the MBD method.^63,75,76^ Furthermore, through the El-QDO model it could also be possible to treat covalent bonds between environment and electronic subsystem following the procedures applied in QM/MM methods^130^ or by modifying the parametrization of the QDOs as a function of inter-QDO distances as suggested in ref (59). Another possible direction would be that of modifying the one-body potential of the drudons in the Coulomb interacting QDOs by defining a repulsive 1/*r^n^* term, with a given radius, used to model the repulsive behavior of true molecular systems.

In conclusion, we have shown how the El-QDO embedding technique offers a unique advantage by incorporating quantum effects for both the subsystem (electrons) and the environment (QDOs) within a framework of quantum-correlated particles. Importantly, the computational cost associated with the environment scales quadratically with its size, as detailed also in ref (57). This quadratic scaling enables the investigation of collective effects, such as the influence of long-range noncovalent interactions from large environments on the polarization, structural properties, and electronic excitations of embedded electronic subsystems with both accuracy and computational feasibility.

## Supporting Information Available

Extended description of the damping functions with a schematic representation; and the results for different types of damping functions and different values of the damping parameter (PDF)
