# Supplementary material for: Molecule–Environment Embedding with Quantum Monte Carlo: Electrons Interacting with Drude Oscillators
Source: J Chem Theory Comput. 2025 Apr 30;21(9):4466–80. doi: 10.1021/acs.jctc.5c00108 (PMC12080123; doi:10.1021/acs.jctc.5c00108)
Supplement: Supplementary file 1 — ct5c00108_si_001.pdf [file ct5c00108_si_001.pdf]

Supporting Information: Molecule–Environment Embedding with Quantum Monte Carlo: Electrons Interacting with Drude Oscillators

Matej Ditte,[1] Matteo Barborini,[1,2] and Alexandre Tkatchenko[1,*]

[1]*Department of Physics and Materials Science, University of Luxembourg, L-1511 Luxembourg City, Luxembourg*

[2]*HPC Platform, University of Luxembourg, L-4364 Esch-sur-Alzette, Luxembourg*

(Dated: April 25, 2025)

* Corresponding author:alexandre.tkatchenko@uni.lu

I. UNIFORM DAMPING

A problem with these standardly used damping functions in QM:MM methods or for systems of interacting QDOs is that they can be interpreted as the charge is a function of the distance between these particles. We will use V_{erf} as an example, but all the following reasoning applies to all other damping functions in Eqs.7-10. V_{erf} can be written as

$$V_{\mathrm{erf}}\left(r_{ij}, \sigma_{ij}\right) = \frac{q_i q_j}{r_{ij}} \mathrm{erf}\left(\frac{r_{ij}}{\sqrt{2}\sigma_{ij}}\right) = \frac{q_i\left(r_{ij}\right) q_j\left(r_{ij}\right)}{r_{ij}}, \tag{1}$$

where $q_i\left(r_{ij}\right)$ and $q_j\left(r_{ij}\right)$ are distance dependent charges (see Fig. S1a). This leads to an artificial polarization because the particles see other parts of the system as artificially charged.

In order to inspect the importance of this effect, we construct so-called 'uniform damping': instead of V_{erf} from Eq. 7, we introduce a damping distance r_{damp} which is taken as the QDO center - QDO center distance in the case of a pair of QDOs and QDO center - atomic center of mass for El-QDO pairs and we use it for the damping part of the potential

$$V_{\mathrm{erf}}\left(r_{ij}, \sigma_{ij}, r_{\mathrm{damp}}\right) = \frac{q_i q_j}{r_{ij}} \mathrm{erf}\left(\frac{r_{\mathrm{damp}}}{\sqrt{2}\sigma_{ij}}\right), \tag{2}$$

where σ_i is defined for the entire electronic system (electrons, nuclei) or the entire QDO (drudons, centers, point charges). To make it clearer, we present a schematic of the uniform damping for an electronic system interacting with two QDOs in Fig. S1b.

In this work, we consider the entire electronic system as one fragment for the calculations of the damping distances. This is because we study only small electronic monomers and thus a further generalization is not necessary. In order to generalize this approach for several monomers or chemical groups of complex systems one would have to assign groups of electrons to individual parts of the atomic systems based on distances during the sampling in the quantum Monte Carlo algorithms.

II. ADDITIONAL RESULTS

Figs. S2-S7 contain the results presented in the main text, extended by more values of sigmas, showing all types of damping functions for the water dimer and the uniform damping for three of the studied systems.

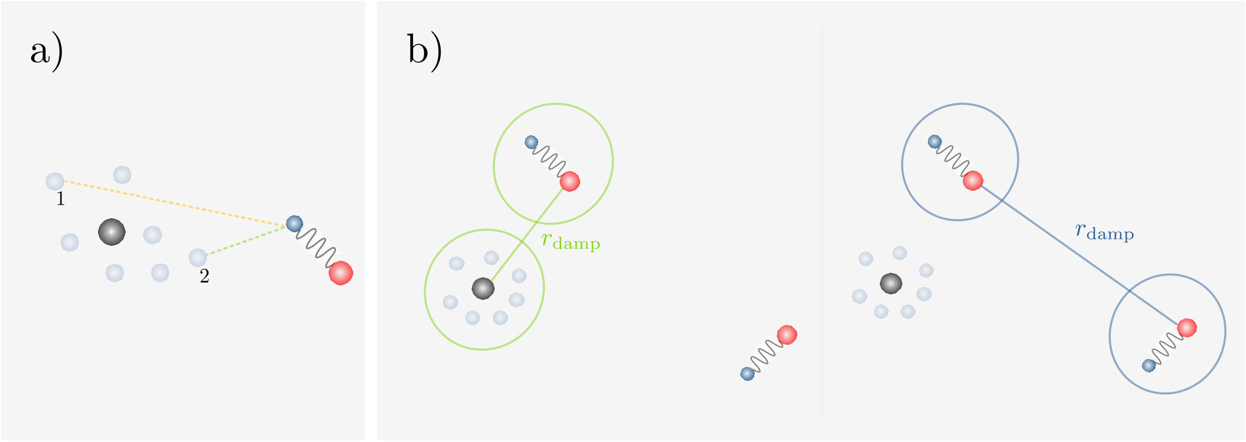

Figure S1. a) Artificial polarization problem due to the standard damping functions, because electron 1 and electron 2 see the drudon as if it had a different charge; b) a schematic of the uniform damping, where the straight lines represent the damping distances r_{damp}, used for the damping of the Coulomb potential between the pairs of particles from circles of the same color.

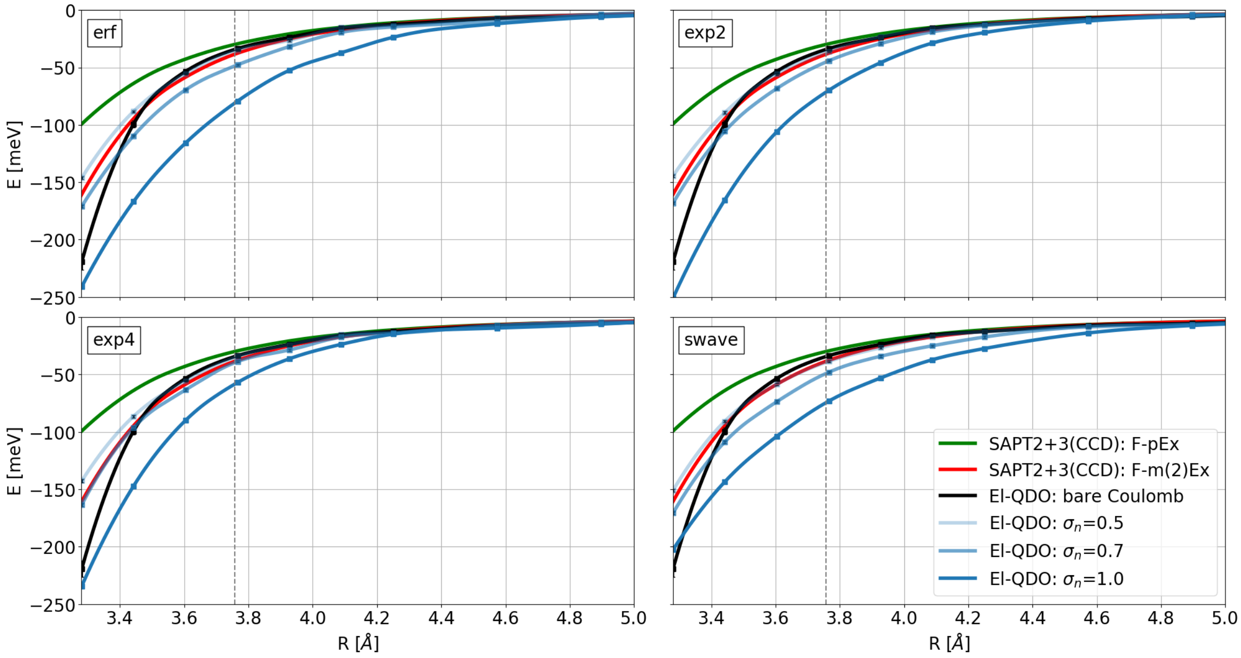

Figure S2. Dissociation of El-QDO argon dimer for erf, exp2, exp4 and swave normal damping functions. The results are compared to the SAPT decompositions explained in the main text.

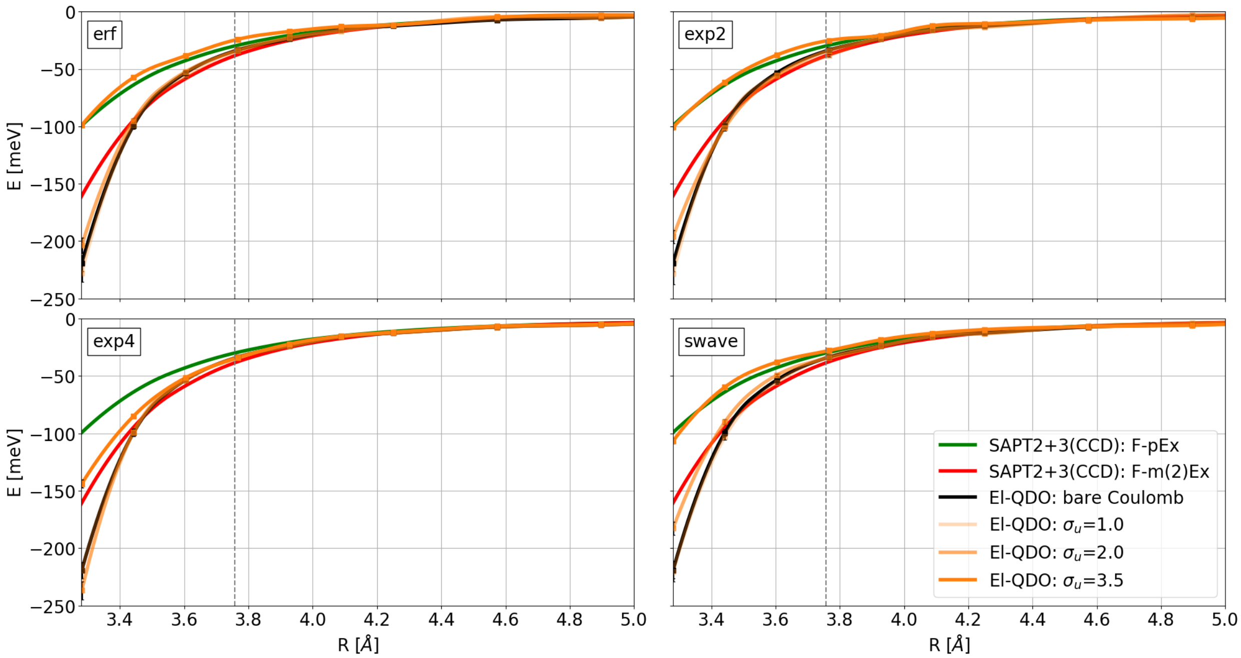

Figure S3. Dissociation of El-QDO argon dimer for erf, exp2, exp4 and swave uniform damping functions. The results are compared to the SAPT decompositions explained in the main text.

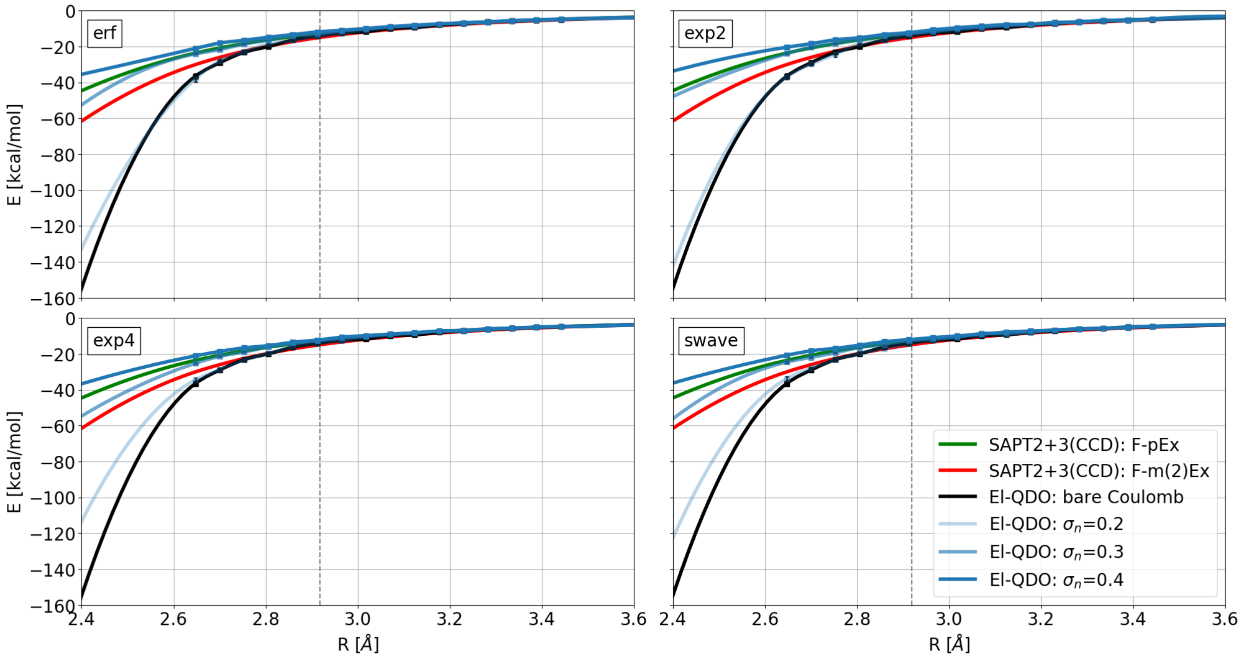

Figure S4. Dissociation of El-QDO water dimer for erf, exp2, exp4 and swave normal damping functions. The results are compared to the SAPT decompositions explained in the main text.

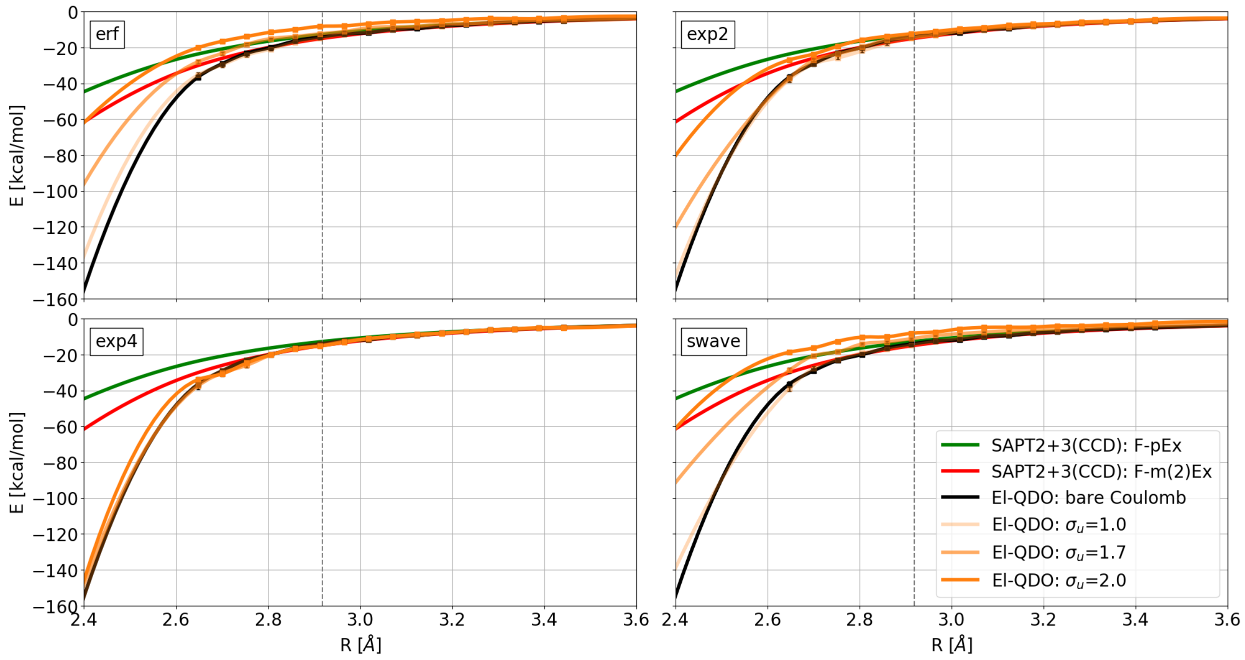

Figure S5. Dissociation of El-QDO water dimer for erf, exp2, exp4 and swave uniform damping functions. The results are compared to the SAPT decompositions explained in the main text.

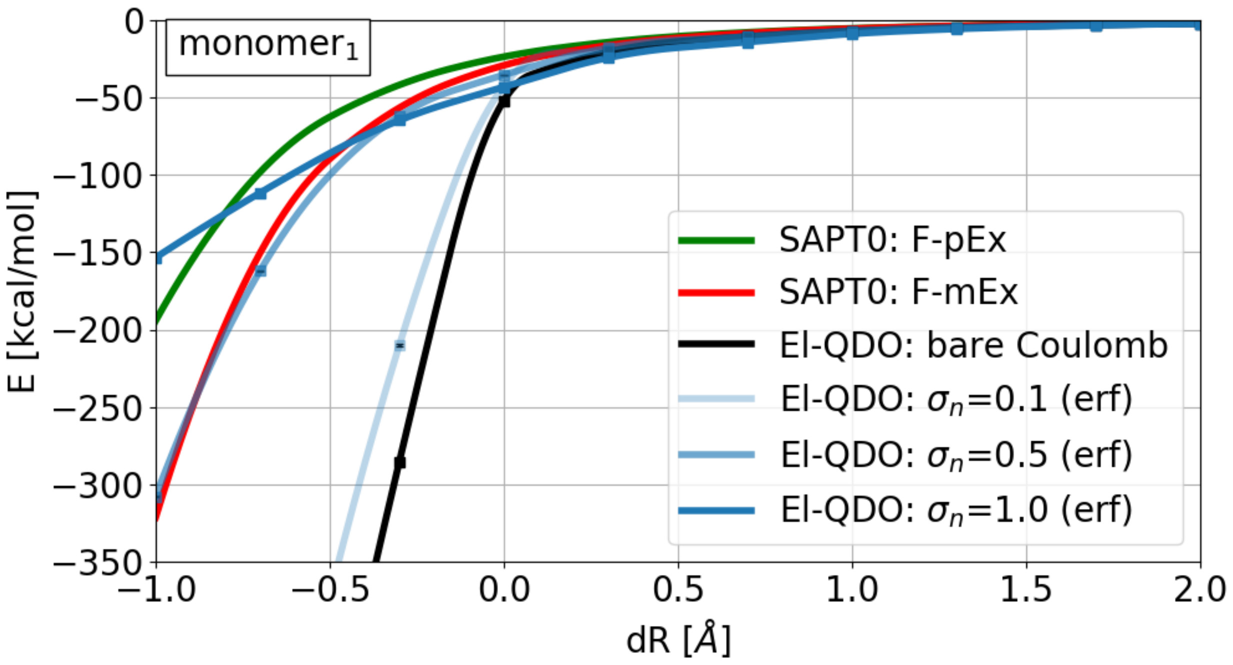

Figure S6. Interaction energies of benzene (monomer$_1$) and an environment composed of 50 water molecules as a function of the expansion of the water cage from its center. El-QDO results obtained with normal damping are compared to SAPT0 the two decomposition explained in the main text.

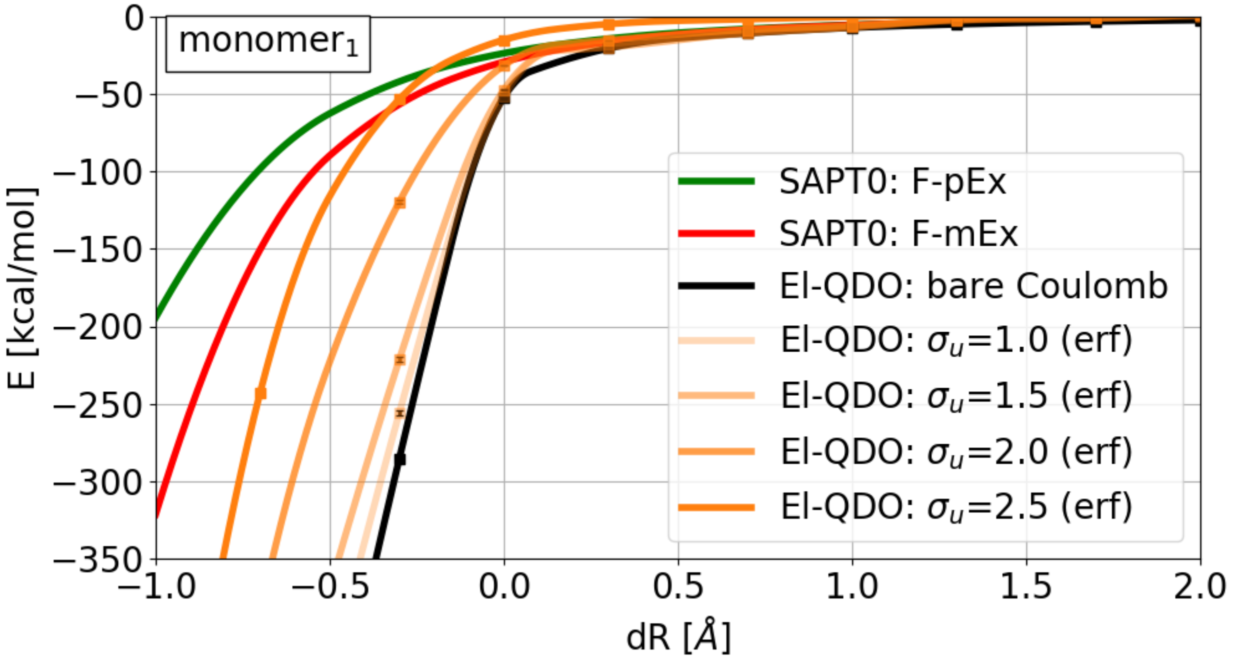

Figure S7. Interaction energies of benzene (monomer$_1$) and an environment composed of 50 water molecules as a function of the expansion of the water cage from its center. El-QDO results obtained with uniform damping are compared to the two SAPT0 decomposition explained in the main text.
